# Correction to ‘Photocatalytic inactivation of *Escherichia coli* under UV light irradiation using large surface area anatase TiO_2_ quantum dots’

**DOI:** 10.1098/rsos.192176

**Published:** 2020-01-22

**Authors:** Faheem Ahmed, Chawki Awada, Sajid Ali Ansari, Abdullah Aljaafari, Adil Alshoaibi

*R. Soc. open sci.* **6**, 191444 (Published Online 4 December 2019). (doi:10.1098/rsos.191444)

The title of this article contained a spelling error. ‘*Escherischia coli*’ should have been ‘*Escherichia coli*’. The incorrect spelling appeared also a number of times in the main text of the manuscript. We apologize for this oversight. This has now been corrected online.

